# Sub-Nanomolar Sensitivity of Nitric Oxide Mediated Regulation of cGMP and Vasomotor Reactivity in Vascular Smooth Muscle

**DOI:** 10.3389/fphar.2012.00130

**Published:** 2012-07-12

**Authors:** Kara F. Held, Wolfgang R. Dostmann

**Affiliations:** ^1^Department of Pharmacology, College of Medicine, University of VermontBurlington, VT, USA

**Keywords:** nitric oxide, cGMP, vasodilation, soluble guanylyl cyclase, phosphodiesterase

## Abstract

Nitric oxide (NO) is a potent dilator of vascular smooth muscle (VSM) by modulating intracellular cGMP ([cGMP]_i_) through the binding and activation of receptor guanylyl cylases (sGC). The kinetic relationship of NO and sGC, as well as the subsequent regulation of [cGMP]_i_ and its effects on blood vessel vasodilation, is largely unknown. In isolated VSM cells exposed to both pulsed and clamped NO we observed transient and sustained increases in [cGMP]_i_, with sub-nanomolar sensitivity to NO (EC_50_ = 0.28 nM). Through the use of pharmacological inhibitors of sGC, PDE5, and PKG, a comprehensive VSM-specific modeling algorithm was constructed to elucidate the concerted activity profiles of sGC, PDE5, phosphorylated PDE5, and PDE1 in the maintenance of [cGMP]_i_. In small pressure-constricted arteries of the resistance vasculature we again observed both transient and sustained relaxations upon delivery of pulsed and clamped NO, while maintaining a similarly high sensitivity to NO (EC_50_ = 0.42 nM). Our results propose an intricate dependency of the messengers and enzymes involved in cGMP homeostasis, and vasodilation in VSM. Particularly, the high sensitivity of sGC to NO in primary tissue indicates how small changes in the concentrations of NO, irrespective of the form of NO delivery, can have significant effects on the dynamic regulation of vascular tone.

## Introduction

Nitric oxide (NO) is produced in small puffs by the nitric oxide synthase of endothelial cells (eNOS), which diffuses into vascular smooth muscle (VSM) and acts directly on the NO-sensitive guanylyl cyclases α1β1/α2β1 (Arnold et al., 1977; Moncada et al., 1991a; Lowenstein and Michel, 2006). The resulting synthesis of cyclic-3′,5′-guanosine monophosphate (cGMP) is critical in mediating vasodilation through activation of cGMP-dependent protein kinase (PKG), which subsequently lowers intracellular calcium (Waldman and Murad, 1987; Sausbier et al., 2000; Schlossmann et al., 2000; Friebe and Koesling, 2003).

The short biological half-life of NO (Griffith et al., 1984; Cocks et al., 1985; Hakim et al., 1996; Wood et al., 2011) has previously served to explain the transient in nature of NO-induced vasorelaxation (Ignarro et al., 1987, 1988; Palmer et al., 1987; Amezcua et al., 1988; Moncada et al., 1991b). However, the kinetics of the NO/cGMP/PKG signaling mechanism has not yet been described in VSM. It has been recently shown that the coupling of NO donors to the NO-scavenger CPTIO alters the kinetics of NO as to deliver a sustained application of NO, which can be modeled to determine NO concentration, previously confirmed by NO electrode (Bellamy et al., 2002; Griffiths et al., 2003). This approach allowed for the direct vascular response to NO to be determined without interference of NO decay. Combination of this NO delivery method with the single wavelength cGMP biosensor, FlincG (Nausch et al., 2008), has recently been described as an exceedingly sensitive NO detector in intact cells, using cGMP as a gauge of sGC activity (Batchelor et al., 2010; Wood et al., 2011). Sub-nanomolar concentrations of NO were detected, albeit dependent on sGC expression level, providing insight into the mechanism for the biological effects of NO at very low concentrations (Batchelor et al., 2010). While this cellular model provides invaluable insight into the NO-dependent activation of sGC, a generalization to all types of mammalian tissues remains controversial. Particularly VSM cells appear to harbor their unique sets of regulatory enzymes in the maintenance of cGMP homeostasis.

A model has also been developed to predict the activities of sGC and phosphodiesterases (PDEs), the two main enzymes involved in cGMP maintenance, in platelets and cerebellar astrocytes (Moncada et al., 1991a; Conti et al., 1995; Francis et al., 2001; Derbyshire and Marletta, 2009; Halvey et al., 2009; Kemp-Harper and Schmidt, 2009). This approach provided an intimate view into the enzymatic control of cGMP production and degradation in platelets and astrocytes. Through a modification of the original model we could determine the activity of endogenous sGC to NO and the level of phosphodiesterases involved in smooth muscle cells of the vasculature.

Here we have found a very high sensitivity to NO in isolated VSM cells as compared to other endogenous sGC activities reported, conforming to a low expression level of sGC. Through the use of the cGMP biosensor δ-FlincG, the regulation of NO-induced [cGMP]_i_ was determined to be through the concerted actions of sGC and PDEs in VSM cells. Likewise, the low EC_50_ for NO was confirmed in pressurized arteries from the resistance vasculature, suggesting the regulation of cGMP directly transcends to the control of vasodilation. This two-pronged *in vitro* and *ex vivo* approach enabled us to establish of the endogenous kinetic inter-relationship of NO, [cGMP]_i_, and vasodilation at physiologically relevant NO concentrations, specific for SM tissue of the vasculature.

## Experimental Procedures

### Materials

The NO donors: DEA/NO, DETA/NO, MAHMA/NO, Spermine/NO, and PROLI/NO were purchased from Cayman Chemical (Ann Arbor, MI, USA). 2-(4-carboxyphenyl)-4,5-dihydro-4,4,5,5-tetramethyl-1H-imidazol-1-yloxy-3-oxide (CPTIO) and 1H-[1,2,4] Oxadiazolo [4,3-a] quinoxalin-1-one (ODQ) were purchased from Sigma-Aldrich (St. Louis, MO, USA). DT-2 was synthesized by Dr. Jose Madalengoitia’s laboratory. Hank’s Balanced Salt Solution (HBSS), DMEM, and penicillin/streptomycin were purchased from Mediatech (Manassas, VA, USA) and Bovine Growth Serum (BGS) was from Hyclone (Logan, UT, USA). Collagenase Type 2 and Elastase were from Worthington Biochemical (Lakewood, NJ, USA).

### Vascular smooth muscle cell culture

Smooth muscle cells from the thoracic aorta of Sprague-Dawley rats were digested, dissociated, and plated on Delta T4 dishes (Bioptechs, Butler, PA, USA) as previously described (Cawley et al., 2007). Cells were minimally cultured (without passaging) before overnight transfection with 85 μL 10^7^–10^9^/mL titer adenoviral δ-FlincG (Nausch et al., 2008) and imaged on the fourth day, unless stated otherwise. Adenoviral constructs were propagated and maintained according to manufacturer’s instructions (Invitrogen). All animal studies and procedures were approved by the Institutional Animal Care and Use Committee at the University of Vermont.

### Fluorescence imaging of cGMP in VSM cells

Live-cell, epi-fluorescent microscopy was performed as previously described (Nausch et al., 2008) in imaging buffer [10 mM TES (pH 7.4), 1 g/L d-glucose, HBSS] using a Nikon Diaphot 200 microscope outfitted with a Nikon x40/1.30 oil objective, mercury-halide lamp (X-CITE 120; EXFO Photonics, Toronto) and a cooled charge-coupled device camera (ORCA ER; Hamamatsu, Japan) capturing one image per 3 s. Confocal imaging was executed on a Nikon E600SN microscope adapted with an Andor spinning disk confocal system, 60× water dipping objective (N.A. 1.0) and iXon ENCCD DVB camera set to acquire five images per second with a 64 ms exposure to a 488 nm solid-state laser. Emission above 510 nm was collected for measurements. Both microscopes used the Delta T4 open culture system to maintain 37°C for the duration of the experiments. NO donors and ODQ stocks were made fresh on the day of use. All compounds were added directly to the imaging buffer and mixed by careful pipetting to avoid cell disturbances and movement. Confocal data analysis was conducted with custom-written software (SparkAn) developed by Dr. Adrian Bonev, while epi-fluorescent analysis was performed using Metafluor version 6.2 software (Universal Imaging, Media, PA, USA; Nausch et al., 2008). All FlincG traces are represented as the ratio of fluorescence signal intensity and background fluorescence (F/F_0_). The mean value reported is a composite of traces from several individual cells digested from multiple aortas. Cellular movement during the course of the experiment was accounted for during analysis. Several small regions of throughout the cell were drawn with imaging software and repositioned if necessary to obtain fluorescent quantifications. A cell is represented by the average of these regions. Dose-response curves were calculated with GraphPad Prism software (version 5.04) for each individual experiment, and then averaged to obtain the mean and standard deviation values.

### Mathematical modeling of NO and cGMP concentrations, PDE and sGC activity

The NO delivery model was developed through differential equations based on the chemical reactions for NO release from NONOate donors and NO consumption by CPTIO and O_2_, as previously described (Griffiths et al., 2003; Roy and Garthwaite, 2006). Tandem to these calculations, formulations for sGC activity were described using the following equations, based on the model of NO binding to the heme of sGC (Halvey et al., 2009). PDE5 activity was solved for by assuming cGMP hydrolysis via four states of the enzyme: unliganded (PDE5, PDE5a), cGMP-bound inactive (cGMP-PDE5, PDE5b), cGMP-bound active (cGMP-PDE5, PDE5c), and phosphorylated (cGMP-P-PDE5*, PDE5d) from the following model [adapted from (Halvey et al., 2009)]:

(1) $$\;\begin{array}{ccccccc} & kp_{1} & & Kp_{2} & & Kp_{3} & \\ PDE5 & ⇄ & cG-PDE\;5 & ⇄ & cG-PDE\;5^{*} & ⇄ & cG-P-PDE\;5^{*}\\ Vp_{1} & kp_{-1} & Vp_{1} & kp_{-2} & Vp_{2} & kp_{-3} & Vp_{3}\\ Kp_{1} & & Kp_{1} & & Kp_{2} & & Kp_{3} \end{array}$$

Activation rates are represented in lower case italicized letters, while catalysis rates are capitalized and italicized in Eq. 1 and below. The rate of cGMP synthesis by NO-activated sGC is represented by *v1(t)* and PDE1 activity is denoted as *Vp*_*x*_ and affinity as *Kp*_*x*_.

The rates are described by the following equations:

$$\begin{array}{rcll} \frac{d}{dt}G\left(t\right) & =v1\left(t\right)-PDE5a\left(t\right)\cdot \frac{Vp_{1}\cdot G\left(t\right)}{Kp_{1}+G\left(t\right)} & & \\ & \;-PDE5b\left(t\right)\cdot \frac{Vp_{1}\cdot G\left(t\right)}{Kp_{1}+G\left(t\right)} & & \\ & \;-PDE5c\left(t\right)\cdot \frac{Vp_{2}\cdot G\left(t\right)}{Kp_{2}+G\left(t\right)} & & \\ & \;-PDE5d\left(t\right)\cdot \frac{Vp_{3}\cdot G\left(t\right)}{Kp_{3}+G\left(t\right)}-\frac{Vp_{x}\cdot G\left(t\right)}{Kp_{x}+G\left(t\right)} & & \\ & & & \text{(2)} \end{array}$$

$$\begin{array}{rcll} \frac{d}{dt}PDE5a\left(t\right) & =kp_{-1}\cdot PDE5b\left(t\right)-kp_{1}\cdot PDE5a\left(t\right)\cdot G\left(t\right) & & \text{(3)} \end{array}$$

(4) $$\begin{array}{c} \frac{d}{dt}PDE5b(t)=kp_{1}\cdot PDE5a(t)\cdot G(t)-kp_{-1}\cdot PDE5b(t)\\ +kp_{-2}\cdot PDE5c(t)-kp_{2}\cdot PDE5b(t) \end{array}$$

(5) $$\begin{array}{c} \frac{d}{dt}PDE5c(t)=kp_{2}\cdot PDE5b(t)-kp_{-2}\cdot PDE5c(t)\\ +kp_{-3}(t)\cdot PDE5d(t)\\ -kp_{3}\cdot G(t)\cdot PDE5c(t) \end{array}$$

(6) $$\frac{d}{dt}PDE5d(t)=kp_{3}*PDE5c(t)-kp_{-3}*PDE5d(t)$$

where *G* denotes cGMP concentration. All differential equations were solved using the Adams/BDF and adaptive Runge–Kutta algorithms in Mathcad (see Supplementary Material; version 14.0; Parametric Technology Corporation, Needham, MA, USA). The velocity and rate constants for the inactive and active NO and cGMP receptors (sGC, PDE5, P-PDE5, and PDE1, respectively), were determined assuming G(0) = 4.16 nM, PDE5a(0) = 0.8, PDE5b(0) = 0, PDE5c(0) = 0, and PDE5d(0) = 0.2 at time zero. Values from previously studies (*Vp_1_*, *Kp_1_*, *Kp_2_*, *Vp_3_*, *Kp_3_*, *Vp_*x*_*, and *Kp_*x*_*; see Table 3) and as determined (GC_max_ and *Vp*_2_) were calculated by the Levenberg-Marquardt method of least squares curve fitting of the proposed model to actual data using the MinErr function of MathCad, as shown in the Supplementary Material and previously described (Wood et al., 2011). The resulting values were then combined to determine the velocity of cGMP hydrolysis (*Vd*) by solving:

$$\begin{array}{rcll} Vd\left(t\right) & =PDE5a\left(t\right)\cdot \frac{Vp_{1}\cdot G\left(t\right)}{Kp_{1}+G\left(t\right)}+PDE5b\left(t\right)\cdot \frac{Vp_{1}\cdot G\left(t\right)}{Kp_{1}+G\left(t\right)} & & \\ & \;+PDE5c\left(t\right)\cdot \frac{Vp_{2}\cdot G\left(t\right)}{Kp_{2}+G\left(t\right)}+PDE5d\left(t\right)\cdot \frac{Vp_{3}\cdot G\left(t\right)}{Kp_{3}+G\left(t\right)} & & \\ & \;+\frac{Vp_{x}\cdot G\left(t\right)}{Kp_{x}+G\left(t\right)} & & \text{(7)} \end{array}$$

The FlincG response to the total calculated cGMP concentration was also modeled based on the binding affinity of cGMP for δ-FlincG (170 nM; Nausch et al., 2008) using the following equation:

(8) $$FG\left(t\right)=\left[\frac{R_{\max}*G{\left(t\right)}^{p}}{G{\left(t\right)}^{p}+{\left(0.17*10^{-6}\right)}^{p}}\right]$$

where *FG(t)* is the predicted FlincG response, *R*_max_ is the maximal percent response of the indicator, and *p* is the Hill coefficient 1.45, as determined from the cGMP binding curves (Nausch et al., 2008). The final algorithm was compiled using the averaged calculated MinErr values for each of the NONOate donors simulated.

### Myography

Anterior cerebral arteries from male Sprague-Dawley rat and third order mesenteric arteries from male mice (~100 μm starting diameter) were isolated and cannulated in physiological saline solution (PSS; 119 mM NaCl, 4.7 mM KCl, 24 mM NaHCO_3_, 1.2 mM KH_2_PO_4_, 0.03 mM EDTA, 1.2 mM MgSO_4_, 1.6 mM CaCl_2_, 10.6 mM glucose, pH 7.4 at 4°C) as previously described in a DMT organ culture myograph (Danish Myo Technology A/S). Warmed (37°C) and gassed (20% O_2_/5% CO_2_/75% N_2_) PSS was circulated through the myograph chamber and arterial diameters were measured using edge detection software (VediView, DMT; Nickl et al., 2010). Intraluminal pressure was progressively increased in 20 mmHg steps using a pressure manometer to allow for the development of myogenic tone (usually between 60 and 80 mmHg) before each experiment. Arterial viability was tested with 60 mM KCl (less than 30% constriction were discarded), and maximal (passive) arterial diameter was obtained for each artery by Ca^2+^-free PSS containing EGTA (2 mM) at the end of each experiment. The NO-scavenger CPTIO was added to the reservoir and allowed to equilibrate before the addition of each NO donor. Donors were added to either the circulating PSS reservoir or the mounting chamber with similar results. Blue-dyed PSS was used to measure the time required for compounds in the reservoir to reach the myograph chamber for kinetic calculations (100 s). The mean value for each experiment refers to separate applications of NO to multiple individual arteries. EC_50_ values were calculated with GraphPad Prism software for each individual artery, and then averaged to obtain mean and standard deviation values.

## Results

### Kinetic profiles of NO and cGMP in VSM cells

**Figure 1:**
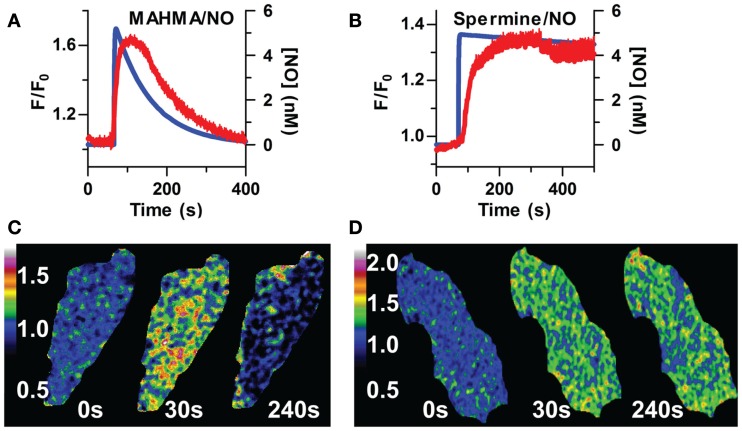
**NO delivery and [cGMP]_i_ profiles are correlated in isolated FlincG-transfected VSM cells**. Application of 5 nM of NO was applied to VSM cells, resulting in transient or sustained [cGMP]_i_ (blue line, modeled NO concentration; red line, F/F_0_ averaged FlincG measured cGMP). **(A)** MAHMA/NO (*n* = 8), **(B)** Spermine/NO (*n* = 9). Distribution of [cGMP]_i_ in response to NO, both transient **(C)** and sustained **(D)**, is globally dispersed throughout the cytoplasm (60× magnification, see Methods).

**Figure 2:**
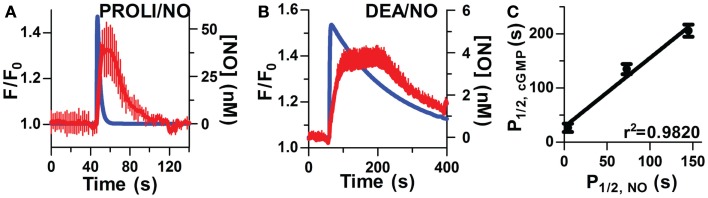
**Kinetic correlation of pulsed NO and [cGMP]_i_**. Short half-life donors result in rapid NO (blue lines, modeled NO concentration) and cGMP peaks (averaged traces, red lines, *n* = 6) as depicted in **(A**; PROLI/NO) and **(B**; DEA/NO). **(C)** Relationship between NO and cGMP transients as measured by *P*_1/2_ (r^2^ = 0.9820, *p* < 0.0001 by one-way ANOVA).

First, we addressed the kinetic nature of intracellular cGMP in primary, δ-FlincG-transfected rat aortic VSM cells to either pulsed (transient) or clamped (sustained) NO profiles by combining NONOate donors with the NO-scavenger, CPTIO (Griffiths et al., 2003). The short half-life donors, PROLI-NO, MAHMA-NO, and DEA-NO (Table 1), gave rise to transient pulses of NO (Figures 1A and 2A,B). Importantly, these transient decreases in cGMP were observed in the absence of buffer washouts to terminate NO exposure, indicating the rate of NO loss was directly dictated by the NO donor half-life. The maximal or peak NO concentrations for each donor were individually calculated based on the rates of NO release as previously described (Griffiths et al., 2003). As shown in Figure 1A, single applications of 200 nM MAHMA/NO in the presence of 50 μM CPTIO gave rise to NO pulses with set peak concentrations of 5 nM (blue trace). The corresponding biosensor responses indicating (cGMP)_i_ (red trace) were equally transient in nature, although we observed a slight lag in the timing of the cGMP responses (2 s vs. 156 s for MAHMA/NO and Spermine/NO, respectively). In contrast, when NO delivery was altered to a clamped pattern using the long half-life donor Spermine-NO (8 + 60 μM CPTIO) the resulting 5 nM NO plateau was echoed by a sustained cGMP profile (Figure 1B). To ensure the addition of CPTIO does not affect cGMP sub-cellular localization, δ-FlincG-transfected VSM cells were imaged by live-cell, fluorescence confocal microscopy. Both the pulsed and clamped conditions (Figures 1C,D) exhibited global distribution, ensuring the addition of the NO-scavenger does not alter the spatial patterning of [cGMP]_i_ in accordance with previous results (Nausch et al., 2008).

**Table 1:** **NO donor and cGMP kinetics**.

Donor	*t*_1/2_ (s)	NO P_1/2_ (s)	cGMP P_1/2_ (s)	τ (s)	*n*
PROLI/NO	1.8	4.5	26.7 ± 7.7	23 ± 6.2	9
MAHMA/NO	63	73	135 ± 9.4	75 ± 8.8	11
DEA/NO	126	145	206 ± 11.3	99 ± 8.1	10
Spermine/NO	2,340	n/a	n/a	n/a	12
DETA/NO	72,000	n/a	n/a	n/a	7

*Summary table of the NO donor half-life (t_1/2_), the NO peak width at half-maximal stimulation (NO P_1/2_), as well as the cGMP peak width at half-maximum (cGMP P_1/2_), cGMP peak decay rate (τ) as measured by δ-FlincG, and number of replicates is given for each NO donor. Values are represented as mean ± standard deviation*.

To further examine the inter-relationship of cGMP and NO kinetics, two additional donors (PROLI/NO and DEA/NO) with widely varying half-lives (Table 1), were used to generate corresponding profiles of NO transients (Figures 2A,B). Analysis of the kinetic parameters (cGMP peak widths and NO dissociation constants) for each donor (PROLI/NO, MAHMA/NO, and DEA/NO), revealed that the cGMP and NO peaks followed a linear correlation (*r*^2^ = 0.9820; Figure 2C). These results further indicate a strong dependency of intracellular cGMP dynamics on the NO release pattern.

### Endogenous sGC is highly sensitive to NO in VSM cells

**Figure 3:**
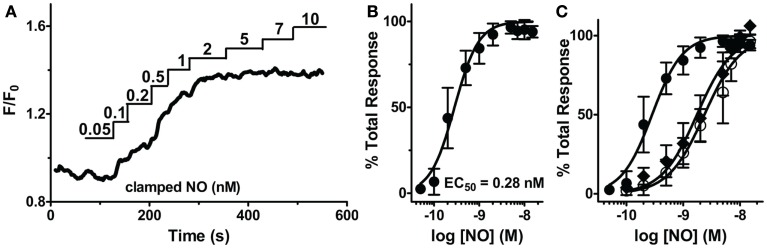
**Sensitivity of sGC to steady-state NO in VSM cells**. **(A)** Representative FlincG trace of step-wise concentrations of clamped NO in VSM cells. **(B)** NO dose-response curve derived from **(A**; *n* = 11).**(C)** EC_50_ curves derived from traces of VSM cells cultured for 4 days (closed circles), 6 days (open circles), and 4 day cultured, Angiotensin II-treated cells (1 μM, diamonds).

By exploiting the steady-state properties of Spermine/NO, additive clamped NO in fixed concentrations (0.05–25 nM) were used to elicit distinct step-wise increases of [cGMP]_i_ (Figure 3A). This procedure allowed us to raise cGMP in progressive increments to extrapolate a direct EC_50_ for sGC activation, as well as illustrate maximal activation, and elucidate any potential indicator saturation to ensure an accurate representation of sGC activity. The fluorescence intensity (F/F_0_) was normalized for each NO step to the maximal fluorescence response elicited by NO (25 nM). The resulting dose-response relationship delivered an EC_50_ value of 0.28 ± 0.01 nM (Figure 3B, *n* = 11). This high sensitivity is comparable to low sGC-transfected HEK cells (GC_low_PDE_HEK_) used as NO reporter cells previously reported (Batchelor et al., 2010; Wood et al., 2011). Likewise, this value shows an approximate threefold increase in potency than that previously reported in platelets and astrocytes (Wykes and Garthwaite, 2004; Roy et al., 2008). The additive steps of NO reach maximal cGMP production by 2 μM, which corresponds with the detection limitation of δ-FlincG (Nausch et al., 2008).

To confirm that this low EC_50_ is not an artifact of a maximally stimulated biosensor, our minimally cultured cells (4 days, unpassaged, see Methods, *n* = 11; shown in Figure 3B) were cultured for an additional 48 h and exhibited a shift of the EC_50_ to ~1 nM (Figure 3C, *n* = 12). This shift demonstrates that the biosensor has not saturated at 1 nM NO (Figure 3B) for the 4 day cultured VSM cells, and the measured EC_50_ values are a valid indication of NO-stimulated sGC production of cGMP and not an artifact of the cGMP binding characteristics of δ-FlincG. To address whether this shift was a result of oxidative stress, possibly due to two additional days of ambient air (20% O_2_) exposure, we activated an endogenous mechanism for inducing oxidative stress, Angiotensin II (1 μM, 1–2 h; Griendling et al., 1994; Nakamura et al., 1998). VSM cells cultured for 4 days and treated with Angiotensin II resulted in a shift similar to the 6 day cultured cells (Figure 3C, *n* = 13). Therefore, sGC sensitivity is not hindered by the usage of δ-FlincG, and the control of cell culture conditions is a vital component to studying cellular NO.

### The concerted actions of both sGC and PDE5 contribute to [cGMP]_i_ in VSM cells

**Figure 4:**
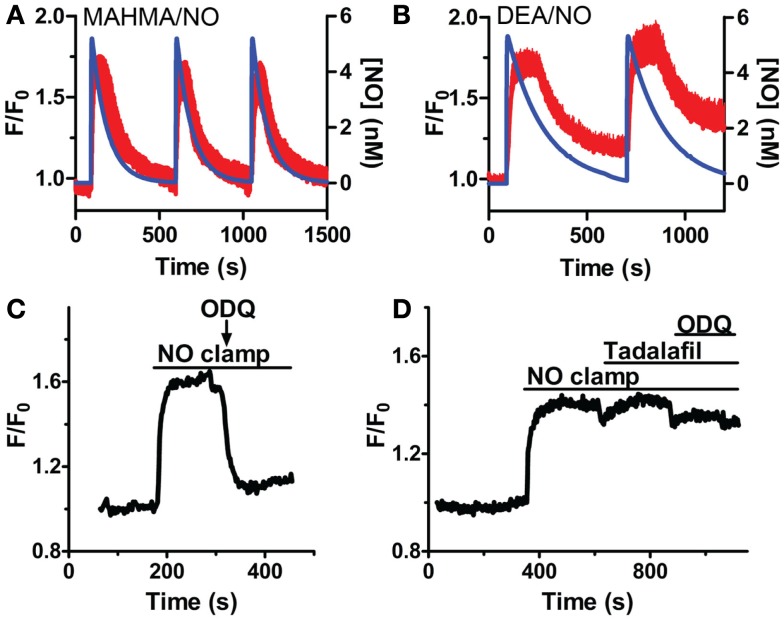
**sGC and PDE5 simultaneously regulate [cGMP]_i_**. Repeated pulses of 5 nM NO using **(A)** MAHMA/NO (200 nM + 50 μM CPTIO, *n* = 5) and **(B)** DEA/NO (400 nM + 50 μM CPTIO, *n* = 6). Shown is the average mean value and standard deviation of FlincG-transfected VSM cells for several experiments in red, calculated NO concentration in blue. **(C,D)** Representative traces depicting decreased [cGMP]_i_ upon 10 μM ODQ application in the presence of clamped 5 nM NO (Spermine/NO, C), *n* = 4 and persistently elevated [cGMP]_i_ upon tadalafil (1 μM) and ODQ (10 μM) treatment, *n* = 7 **(D)**.

While sGCα1/β1 is known to produce [cGMP]_i_ upon NO activation, it is not known if sGC activity will remain elevated throughout a sustained application of NO in VSM cells, or if it rapidly desensitizes as in platelets and neurons in the continued presence of NO (Arnold et al., 1977; Moncada et al., 1991a; Bellamy et al., 2000; Bellamy and Garthwaite, 2001; Kass et al., 2007). Repeated applications of NO in the presence (Figures 4A,B) and absence (Cawley et al., 2007; Nausch et al., 2008) of CPTIO resulted in stable reproducible increases in [cGMP]_i_, with little decrease in their amplitudes, suggesting modest sGC desensitization, and a decrease in sGC activity after NO decay. To determine the duration of sGC activation, the sGC inhibitor, ODQ, was administered in the presence of sustained NO. This resulted in the rapid reduction of [cGMP]_i_, inferring a consistently active sGC, as well as elevated phosphodiesterase activity (Figure 4C). The PDE5-specific inhibitor, tadalafil, was next applied to implicate PDE5 as the major phosphodiesterase in VSM. Despite ODQ inhibition of sGC, [cGMP]_i_ remained elevated in the presence of tadalafil and sustained NO (Figure 4D). We noted that the detection limitation of δ-FlincG prevented any further increase in [cGMP]_i_ fluorescence during the simultaneous inhibition of sGC and PDE5. Nevertheless, since a lack of cGMP degradation was seen in Figure 4D as compared to Figure 4C, it can be concluded that not only is sGC active throughout the entire application of clamped NO, but PDE5 is simultaneously active, thus both enzymes are required for the concerted maintenance of [cGMP]_i_.

### Modeling sGC and PDE activities

**Figure 5:**
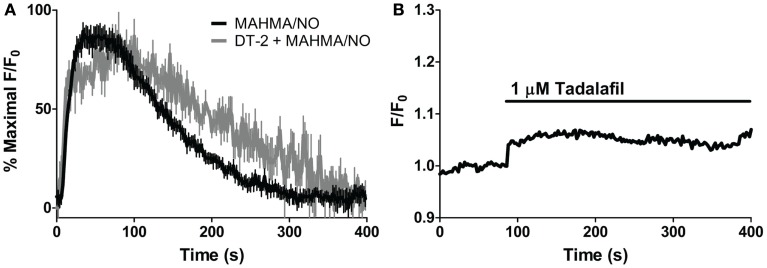
**Effects of PKG and PDE5 inhibition on cGMP**. **(A)** Averaged trace of mean cGMP as measured by FlincG in response to 5 nM NO (MAHMA/NO, black) and the PKG-specific inhibitor, DT-2, treated 5 nM NO peak (1 μM, gray) each normalized to percent maximal F/F0 ratio. **(B)** Application of the PDE5-specific inhibitor, tadalafil (1 μM). Shown is the average mean value, *n* = 8.

In order to elicit the kinetic relationship of sGC and PDE5 to the dynamic applications of NO and cGMP, we modified a recently developed model of cGMP regulation in platelets (Roy and Garthwaite, 2006; Halvey et al., 2009) to specifically reflect VSM cells (Table 3). As sGC and PDE5 have already been implicated as key cGMP regulators (Figure 4), the involvement of phospho-PDE5 (P-PDE5) was scrutinized in regards to the maintenance of VSM [cGMP]_i_. To block the phosphorylation of PDE5, we applied DT-2, a membrane-permeable and specific inhibitor of PKG1α (Dostmann et al., 2000; Nickl et al., 2010). NO-induced cGMP transients (5 nM NO pulse, MAHMA/NO) elicited in the presence of DT-2 were markedly broader (~20–30% increase as measured by peak width and area under the curve; Figure 5A; Table 2), attributing the phosphorylated PDE5 as a key manager of VSM [cGMP]_i_. Phosphorylated PDE5 has been shown by us and others to remain elevated upon NO stimulation for up to 1 h, implicating a possible role for P-PDE5 as a long-term phosphodiesterase (Mullershausen et al., 2001, 2003; Rybalkin et al., 2002; Cawley et al., 2007; Halvey et al., 2009). We have previously shown a small but significant increase in baseline cGMP upon application of the PDE5 inhibitor, sildenafil, suggesting a basal activity of PDE5, likely due to P-PDE5, which may remain active for over an hour (Rybalkin et al., 2002; Cawley et al., 2007). Tadalafil was used here over sildenafil due to the enhanced specificity of tadalafil for PDE5 (Bischoff, 2004). Tadalafil also produced a minor increase in baseline cGMP (4.4 ± 1.1% F/F_0_, *n* = 8; Figure 5B), lending support to the presence of P-PDE5 in the absence of NO exposure.

**Table 2:** **The effect of P-PDE5 on cGMP peaks**.

Donor	cGMP P_1/2_	A.U.C.
MAHMA/NO	135 ± 9.4 (11)	13610 ± 1685 (11)
DT-2 + MAHMA/NO	175 ± 21.4 (6)	16776 ± 2259 (6)
	30% increase	23% increase

*Measurement parameters of MAHMA/NO-induced cGMP peaks with and without P-PDE5 through PKG inhibition by DT-2 by measuring peak width at half-maximal stimulation (cGMP P_1/2_) and area under the curve (A.U.C.)*.

**Figure 6:**
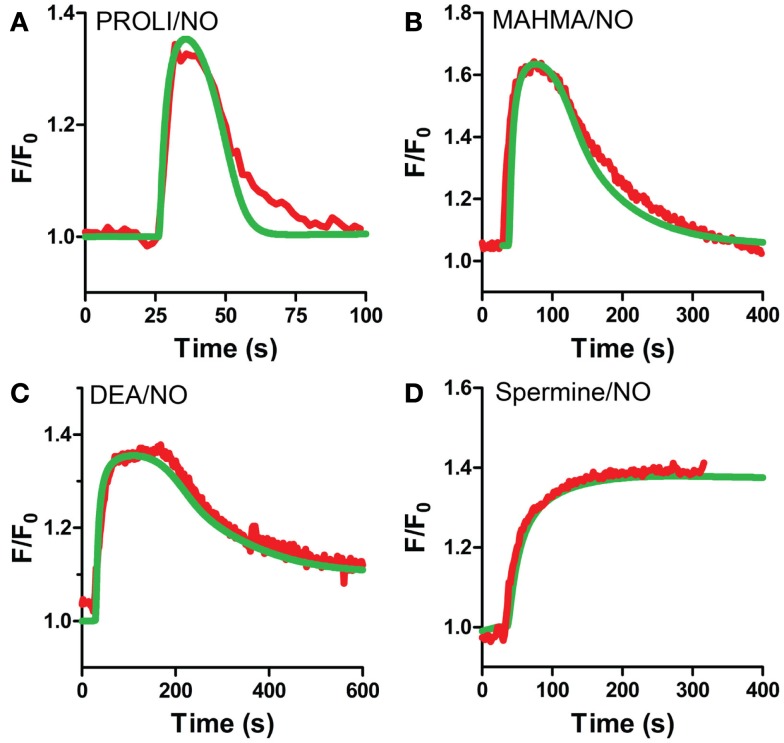
**VSM model predicted cGMP traces for multiple NO donors**. VSM-specific model predicted cGMP (green) and actual cGMP measured by FlincG (red, averaged traces) after exposure to NO for **(A)** PROLI/NO, **(B)** MAHMA/NO, **(C)** DEA/NO, and **(D)** Spermine/NO (FlincG traces are mean values from Figures 2 and 3).

**Figure 7:**
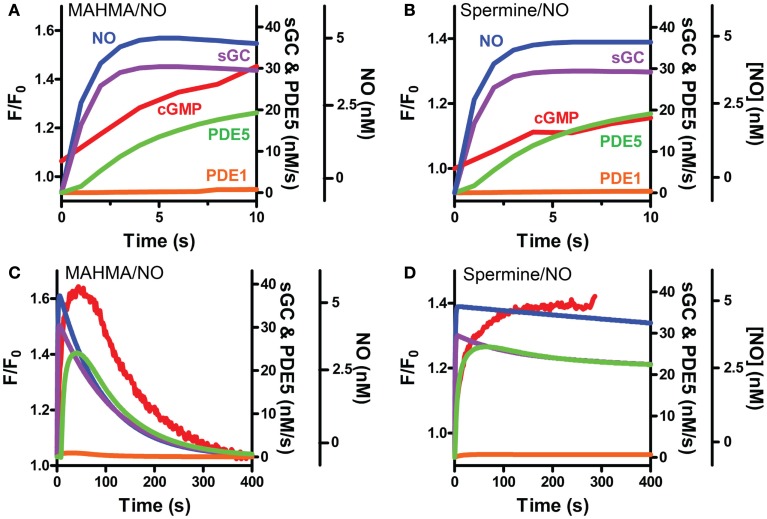
**Modeled sGC, PDE5 and PDE1 activities follow pulsed and clamped NO**. FlincG-monitored cGMP (red) exposed to a 5 nM NO **(A,C)** pulse with MAHMA/NO or **(B,D)** clamp using Spermine/NO are depicted with the corresponding NO concentrations (blue) and modeled enzymatic activities of sGC (purple), PDE5 (green) and PDE1 (orange). Enlargement of the first 10 s for pulsed and clamped NO [**(A)** and **(B)**, respectively].

Our VSM-specific model was determined to require the inclusion of an additional non-cGMP activated PDE, likely PDE1, which has been shown to be present in VSM cells (Rybalkin et al., 1997, 2002), was found to be necessary to account for the rapid degradation phases of cGMP illustrated in Figures 1A and 4A,B (Table 2). The lack of further increase upon tadalafil treatment (Figure 5B) under constant sGC baseline activation also suggested the presence of a second PDE not regulated by cGMP. Finally, a comprehensive model for cGMP modulation in VSM cells was compiled through the use of previously published affinity and activity rates of sGC, PDE5, P-PDE5, and PDE1 (see Table 3) and analysis of our own data to calculate values specific for VSM cells using cGMP peaks elicited by multiple NO donors (Figures 2 and 3), the decay of cGMP following ODQ application (Figure 4C). Values were solved by establishing the minimal sum of squares of the errors between the modeled and experimental cGMP concentrations to solve for the rates of sGC activity as well as phosphorylated and de-phosphorylated PDE5 (Table 3, see Methods). Remarkably, this VSM-specific global model precisely portrays global biosensor-derived cGMP dynamics (Figure 6). Here, the NO donors exemplified by a wide range of NO release rates were equally modeled to portray internal cGMP concentrations as measured by FlincG. The close match of the modeled trace with the measured cGMP supports our model for VSM [cGMP]_i_ dynamics. In addition, the behavior of pulsed (Figures 7A,C) and clamped (Figures 7B,D) NO, spanning a 40,000-fold difference in release rates, are simulated with strong accuracy. Also of note, the enzyme activities determined for the *V*_max_ for sGC and PDE5 (0.19 ± 0.06 μM s^−1^ and 75 ± 3 nM s^−1^, respectively; Table 3) were calculated to be significantly lower than that for platelets [130 and 125 μM s^−1^, respectively (Halvey et al., 2009)]. This relatively low enzyme activity is likely accounted for by a low expression of sGC, similar to that seen in GC_low_PDE_HEK_ cells as previously stated (Batchelor et al., 2010).

**Table 3:** **The mathematical model parameters and values**.

Parameter	Value	Description
*k*_1_	3 × 10^−8^ M^−1^s^−1^	Activation and deactivation constants of sGC, previously determined (Garthwaite, 2005; Roy et al., 2008).
*k*_−1_	6 s^−1^	
*k*_2_, k_−2_	28 s^−1^	
*k*_3_, k_4_, k_5_, k_6_, k_−3_,k_−4_, k_−5_, k_−6_	1000 M^−1^s^−1^, 200 s^−1^, 7.34 × 10^−4^ M^−1^s^−1^, 1 s^−1^, 100 s^−1^, 18 × 10^−5^ M^−1^s^−1^, 4 × 10^−8^ s^−1^, 0.001 s^−1^	sGC desensitization rates (Halvey et al., 2009)
GC_max_	0.19 ± 0.06 μM s^−1^	Maximum sGC velocity
*kp*_1_	1.7 × 10^4^ M^−1^ s^−1^	Binding affinity of cGMP for basal PDE5 (Halvey et al., 2009)
*kp*_−1_	0.13 s^−1^	
*kp*_2_	0.3 s^−1^	Activation rates of catalytically active PDE5 (Halvey et al., 2009)
*kp*_−2_	0.12 s^−1^	
*kp*_3_	0.2 × 10^5^ M^−1 ^s^−1^	Activation rates of phosphorylated PDE5 (Halvey et al., 2009)
*kp*_−3_	2 × 10^−3^ s^−1^	
*Vp*_1_	*Vp*_2_×0.05	Maximum velocity of basal PDE5 (Halvey et al., 2009)
*Vp*_2_	75 ± 3 nM s^−1^	Maximum velocity of active PDE5
*Vp*_3_	2 × *Vp*_2_	Maximum velocity of phosphorylated PDE5 (Corbin et al., 2000)
*Vp_x_*	30 nM s^−1^	Maximum velocity of PDE1
*Kp*_1_	3 μM	Apparent Michaelis constants for basal PDE5, active PDE5, and phosphorylated PDE5, respectively (Corbin et al., 2000; Rybalkin et al., 2003; Halvey et al., 2009).
*Kp*_2_	0.351 μM	
*Kp*_3_	0.5 × *kp*_2_	
*Kp_x_*	1 μM	Apparent Michaelis constant for PDE1 (Bender and Beavo, 2006)

*The parameters, values, and description of each factor used in the mathematical model of sGC and PDE activity of VSM cells*.

### The kinetic profiles of NO formation and vasomotor responses in isolated arteries

**Figure 8:**
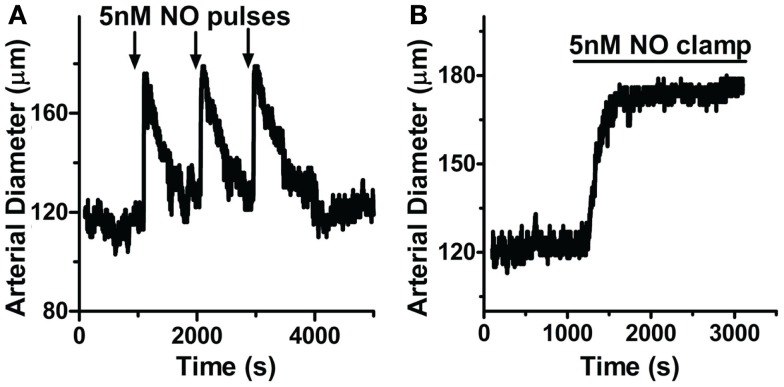
**NO delivery and vasodilation profiles are correlated in pressurized cerebral arteries**. Cannulated arteries under 80 mmHg were treated with **(A)** pulsed NO (200 nM MAHMA/NO + 50 μM CPTIO; *n* = 7) or **(B)** clamped NO (8 μM Spermine/NO + 60 μM CPTIO; *n* = 6), resulting in repeatable transient or sustained relaxations, respectively.

Since NO appears to control a tight regulation on [cGMP]_i_, we next determined if this modulation extends to the regulation of vasomotor reactivity in intact blood vessels. Cannulated arteries from anterior rat brain with 80 mmHg intraluminal pressure were allowed to develop myogenic tone (see Methods). Here, repeated applications of 5 nM NO pulses (200 nM MAHMA/NO + 50 μM CPTIO) elicited transient relaxations of small resistance arteries (Figure 8A; peak width at half-maximum (*P*_1/2_) = 150 ± 26.2 s, *n* = 7; 85 ± 14.5% total maximal relaxation as determined by Ca^2+^-free PSS, *n* = 6; see Methods), similar to those seen in isolated cells (Figures 1A and 2A,B). These transient relaxations were not only elicited in the absence of CPTIO, but also observed using other short half-life donors (DEA/NO and PROLI/NO, data not shown). The similar peak characteristics for NO-induced vasodilation and [cGMP]_i_ (mean *P*_1/2_ = 150 s and 135 s, respectively, Figures 8A and 1A; Table 1) strongly indicate a mechanistic link between the intracellular signaling cascade and the observed physiology. Likewise, arteries exposed to 5 nM clamped NO (8 μM Spermine/NO + 60 μM CPTIO), exhibited a sustained relaxation (75.8 ± 16.5%, maximal relaxation), similar to the [cGMP]_i_ kinetics observed earlier (Figure 8B). Importantly, NO application to either the chamber or circulating buffer of the myograph setup resulted in the same transient or sustained vasodilator responses for either pulsed or clamped NO respectively, indicating these effects are not an artifact of NO washout, and are in fact due to the NO donor release rate (see Methods).

**Figure 9:**
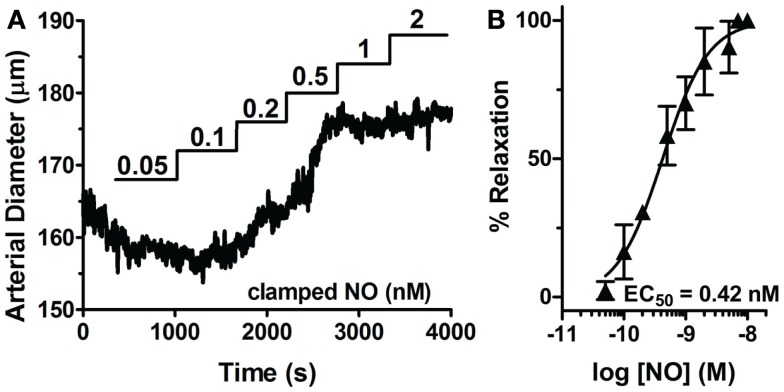
**Sensitivity of sGC to steady-state NO in VSM cells and pressurized mesenteric arteries**. **(A)** Representative myograph trace of a cannulated mesenteric artery with myogenic tone exposed to step-wise increases of clamped NO (0.05–2 μM). **(B)** NO dose-response curve derived from multiple NO exposed arteries (*n* = 5). EC_50_s are calculated from individual myograph traces, then averaged (see Methods).

Through the unique coupling of clamped NO delivery to small pressurized arteries and FlincG-transfected single VSM cells, we report here that the driving force behind vasomotor reactivity is both the dynamic behavior of NO, as well as its exquisitely high sensitivity toward sGC specifically in the vasculature. NO sensitivity of arteries was determined through lumen diameter measurements in response to application of step-wise steady-state NO (0.05 to 25 nM NO, Spermine/NO + 60 μM CPTIO; Figure 9A). The difference of the diameter from baseline for each NO concentration was normalized to maximal NO-stimulated relaxation elicited, and an EC_50_ determined (Figure 9B). The NO-induced dilation EC_50_ was observed as 0.42 ± 0.08 nM (*n* = 5), nearly identical to the value obtained in single VSM cells (0.28 ± 0.01 nM, Figure 3B), suggesting a strong linkage of the kinetics and sensitivity of NO between single cells and whole arteries. To account for the effect of endogenous NO, this myograph experiment was repeated in the presence of the eNOS inhibitor l-NNA, with no change on pulsed NO kinetics or EC_50_ (data not shown). Confirmation of the low EC_50_ in VSM cells also suggests that the traces obtained using the FlincG biosensor are a valid and direct indication of the sensitivity of NO for sGC, and not a titration of cGMP to the indicator.

## Discussion

The spatial and temporal regulation of [cGMP]_i_ has become a recent prime focus in vascular biology (Nausch et al., 2008; Batchelor et al., 2010). As cGMP is a critical mediator for the regulation of vascular relaxation, and therefore blood flow and blood pressure, understanding how [cGMP]_i_ is managed in VSM cells provides invaluable information to the molecular mechanisms underlying vascular physiology. It is widely accepted that the rapid decay of NO explains the previously observed transient dilations in aortic rings (Ignarro et al., 1987, 1988; Palmer et al., 1987; Amezcua et al., 1988; Moncada et al., 1991b). Our work further expands this notion by providing evidence that the enzymes responsible for the regulation of cGMP have adapted to encompass both pulsed and sustained exposure to NO in both intact arteries and single VSM cells (Figures 1, 2, 4, and 8).

Previous attempts to measure sGC potency have used a wide variety of NO and sGC sources such as purified protein and cellular fractions from platelets, aorta homogenates and cerebral fractions using NO donors and gas, resulting in a wide range of EC_50_ values from 0.9 nM to 1.6 μM (Stone and Marletta, 1996; Artz et al., 2001; Bellamy et al., 2002; Wykes and Garthwaite, 2004; Roy and Garthwaite, 2006; Roy et al., 2008). This report documents the first application of clamped NO to elicit an EC_50_ at both the level of individual cells, as well as intact whole tissue in a physiological setting. The threefold increase in sensitivity to the endogenous sGC reported here to that of other cell types (i.e., neurons, platelets, and astrocytes), is likely due to the low level of sGC in VSM as previously demonstrated (Batchelor et al., 2010). GC_low_PDE_HEK_ cells have shown an unprecedented sensitivity to NO, producing relevant concentrations of cGMP for its downstream kinase, PKG (Batchelor et al., 2010). The duplication of this sensitivity seen in VSM cells confirms the need for sub-nanomolar NO activation of GC to provide a physiological response in vascular tissue. The confirmation of a low EC_50_ in isolated VSM cells (Figure 3B) to that of intact arteries (Figure 9), as well as the previously observed EC_50_ of GC_low_PDE_HEK_ cells (Batchelor et al., 2010; Wood et al., 2011), revealed that our isolated cell culture system is a sound reflection of whole tissue physiology. This also suggests that measuring [cGMP]_i_ is a sufficient mechanism to predict the vascular response. Additionally, the low EC_50_ is not a reflection of a titration of the cGMP biosensor, but rather a veritable indication of sGC potency.

The accuracy of the cGMP-sensor FlincG for determining cGMP concentration has been recently questioned, as it can become saturated (Batchelor et al., 2010). δ-FlincG has a dynamic range of cGMP detection of approximately 20 nM to 2 μM (Nausch et al., 2008). Due to the 2–4 μM maximal concentrations of intracellular cGMP (Matchkov et al., 2004), δ-FlincG has proven to be sensitive enough to capture most of the NO response. An induction of [cGMP]_i_ could overcome the maximal detection level of FlincG and no further increase would be observed (as in Figure 4D), yet the temporal dynamics of [cGMP]_i_ below this 2 μM limitation will still be very accurately measured, which has been recently demonstrated (Batchelor et al., 2010; Wood et al., 2011). All critical measurements of FlincG here were used sub-maximally to ensure proper quantification. We are also assured that the biosensor is not saturated through the EC_50_ shift observed in VSM cells under oxidative stress (Figure 3C). Together, this provides further evidence for a dynamic kinetic link between the NO concentration applied and the vasodilatory response achieved, with cGMP acting as a direct intracellular messenger.

While the sensitivity to NO is similar in GC_low_PDE_HEK_ and VSM cells and tissue, the mechanism for cGMP regulation was shown to differ. GC_low_PDE_HEK_ degrade cGMP by the endogenous PDE, PDE1. VSM cells require not only PDE1, but PDE5 and phospho-PDE5 in order to maintain [cGMP]_i_, as suggested by our model and pharmacological studies with the PDE and PKG inhibitors, sildenafil, tadalafil, and DT-2 (Figures 4 and 5). This varies greatly from the model of platelet and astrocyte [cGMP]_i_ in which P-PDE5 does not show a further effect on cGMP degradation and does not require PDE1 (Halvey et al., 2009). Interestingly, the activity of VSM PDEs is twice that of GC_low_PDE_HEK_ (see Table 3), leading to more rapid cGMP transients; a critical action in order to maintain a homeostatic vascular dynamic.

With the high prevalence of vascular diseases such as hypertension, erectile dysfunction and stroke (NIH, 2011; WHOIS, 2011), a full understanding of the kinetic relationship of the NO/cGMP signaling pathway may provide essential therapeutic targets for the treatment, and perhaps prevention, of these diseases.

## Conflict of Interest Statement

The authors declare that the research was conducted in the absence of any commercial or financial relationships that could be construed as a potential conflict of interest.

## Supplementary Material

The Supplementary Material for this article can be found online at http://www.frontiersin.org/Cardiovascular_and_Smooth_Muscle_Pharmacology/10.3389/fphar.2012.00130/abstract
